# Introduction and perspective, historical note

**DOI:** 10.3389/fncel.2013.00227

**Published:** 2013-11-21

**Authors:** Geoffrey Burnstock

**Affiliations:** ^1^Autonomic Neuroscience Centre, University College Medical SchoolLondon, UK; ^2^Department of Pharmacology, The University of MelbourneMelbourne, VIC, Australia

**Keywords:** brain, skin, lung, gut, bladder, cancer, pain, inflammation

## Abstract

P2 nucleotide receptors were proposed to consist of two subfamilies based on pharmacology in 1985, named P2X and P2Y receptors. Later, this was confirmed following cloning of the receptors for nucleotides and studies of transduction mechanisms in the early 1990s. P2X receptors are ion channels and seven subtypes are recognized that form trimeric homomultimers or heteromultimers. P2X receptors are involved in neuromuscular and synaptic neurotransmission and neuromodulation. They are also expressed on many types of non-neuronal cells to mediate smooth muscle contraction, secretion, and immune modulation. The emphasis in this review will be on the pathophysiology of P2X receptors and therapeutic potential of P2X receptor agonists and antagonists for neurodegenerative and inflammatory disorders, visceral and neuropathic pain, irritable bowel syndrome, diabetes, kidney failure, bladder incontinence and cancer, as well as disorders if the special senses, airways, skin, cardiovascular, and musculoskeletal systems.

## Introduction

Division of receptors for purines into P1 (adenosine) and P2 (ATP/ADP) families was proposed in 1978 (Burnstock, 1978). In 1985, P2 receptors were divided into two subtypes, P2X and P2Y receptors, on the basis of pharmacology (Burnstock and Kennedy, 1985). In the early 1990's, P2 receptors for purines and pyrimidines were cloned and characterized and second messenger mechanisms determined (see Abbracchio and Burnstock, 1994; Ralevic and Burnstock, 1998). P2Y_1_ (Webb et al., 1993) and P2Y_2_ (Lustig et al., 1993) G protein-coupled receptors were described initially and soon after P2X1 and P2X2 ion channel receptors were reported (Brake et al., 1994; Valera et al., 1994). Seven P2X receptor subunits have been identified. P2X receptors have been cloned from many eukaryotic species, including mammals, fish, parasitic trematode worms, amoeba, slime mould, and green algae (see Fountain and Burnstock, 2009; Burnstock and Verkhratsky, 2012a). The physiology and pathophysiology of P2X receptors in diseases of the special senses, urinary tract, gastrointestinal tract, pancreas, skin, and musculoskeletal system, as well as in cancer and inflammatory disorders will be discussed.

It was assumed for a long time that the main source of ATP acting on purinoceptors was damaged or dying cells. It is now clear, however, that ATP is released, without causing damage, from many cell types, including endothelial and urothelial cells, macrophages, astrocytes, odontoblasts and osteoblasts, in response to gentle mechanical disturbance, hypoxia, and some agents (Bodin and Burnstock, 2001; Lazarowski et al., 2011; Lazarowski, 2012). Release of ATP initiates purinergic mechanosensory transduction that is involved in bone remodeling (Orriss et al., 2010) and visceral pain via P2X3 receptors on nociceptive sensory nerves (Burnstock, 1999, 2007b). The mechanism of ATP transport from cells appears to be a combination of vesicular exocytosis and connexin and/or pannexin 1 hemichannels (see Lazarowski, 2012). Ectoenzymes are involved in the breakdown of released ATP into ADP, AMP, adenosine, inosine and hypoxanthine (see Zimmermann, 2006; Yegutkin, 2008). These enzymes include NTPDases, pyrophosphatase/phosphodiesterases, alkaline phosphatases, 5′- nucleotidase and monoamine oxidase.

## P2X receptor subtypes

Seven P2X subunits have been cloned and characterized. The P2X1 to P2X6 receptors are 379–472 amino acids long, while the P2X7 receptor is 595 amino acids long, due to the increased length of the COOH terminus. The molecular physiology of P2X receptors has been thoroughly reviewed (see North, 2002). Each subunit possesses two hydrophobic, transmembrane spanning regions that span the cell plasma membrane. A seminal study has been published describing the crystal structure of the P2X4 receptor (Gonzales et al., 2009; Kawate et al., 2009). When P2X7 receptors are occupied by ATP, cation channels are activated, but in addition with high concentrations of ATP large pores are formed which lead to uptake of Ca^2+^ leading to apoptotic cell death.

The seven P2X subtypes combine as trimers (Nicke et al., 1998), which form functional homo- and heteromultimers (see Burnstock, 2007a). P2X6 receptors do not form a homomultimer, while P2X7 receptors do not form a heteromultimer. P2X1/2, P2X1/4, P2X1/5, P2X2/3, P2X2/6, and P2X4/6 heteromultimers have been identified.

## Distribution of P2X receptors

Detailed analyses of the distribution of P2X receptors on nerves and non-neuronal cells have been published (Burnstock and Knight, 2004; Burnstock, 2007b; see Table 1).

**Table 1:** **Principal P2X receptors expressed by excitable tissues and non-neuronal cells (Compiled from Burnstock, 2007b)**.

**NEURONAL**	
Sympathetic neurons	P2X1-7
Parasympathetic neurons	P2X2, P2X3, P2X4, P2X5
Sensory neurons	P2X1-7, predominantly P2X3 and P2X2/3
Enteric neurons	P2X2, P2X3, P2X4, P2X7
Central nervous system	P2X2, P2X4 and P2X6 (perhaps heteromultimers) predominate, (P2X7?)
Retinal neurons	P2X2, P2X3, P2X4, P2X5, P2X7
**MUSCLE CELLS**	
Smooth muscle	P2X1-7, predominantly P2X1
Skeletal muscle	
-Developing	P2X2, P2X5, P2X6
-Adult	P2X1-7
Cardiac muscle	P2X1, P2X3, P2X4, P2X5, P2X6
**NON-NEURONAL**	
Osteoblasts	P2X1, P2X2, P2X5, P2X7
Osteoclasts	P2X1, P2X2, P2X4, P2X7
Cartilage	P2X2
Keratinocytes	P2X2, P2X3, P2X5, P2X7
Fibroblasts	P2X7
Adipocytes	P2X1
Epithelial cells (lung, kidney, trachea, uterus, cornea)	P2X4, P2X5, P2X6, P2X7
Astrocytes	P2X1-7
Oligodendrocytes	P2X1
Microglia	P2X4, P2X7
Müller cells	P2X3, P2X4, P2X5, P2X7
Enteric glial cells	P2X7
Sperm	P2X2, P2X7
Endothelial cells	P2X1, P2X2, P2X3, predominately P2X4
Erythrocytes	P2X2, P2X4, P2X7
Platelets	P2X1
Immune cells (thymocytes, macrophages, neutrophils, eosinophils, lymphocytes, mast cells, dendritic cells)	P2X4 and predominately P2X7, but some P2X1, P2X2, P2X5
Exocrine secretary cells	P2X1, P2X4, P2X7
Endocrine secretory cells (pituitary, pancreas, adrenal, thyroid, testis)	P2X1-7, predominately P2X2/6
Cholangiocytes	P2X2, P2X3, P2X4, P2X6
Interstitial cells of Cajal	P2X2, P2X5
Kupffer cells	P2X1, P2X4, P2X7
Special senses	
Inner ear	P2X1, P2X2, P2X3, P2X7
Eye	P2X2, P2X7
Tongue	P2X2, P2X3
Olfactory organ	P2X2, P2X4
Cochlea hair cells	P2X1, P2X2, P2X7

## Physiology of P2X receptors

ATP released as a cotransmitter with noradrenaline (NA) from sympathetic nerves was shown to act mainly via P2X1 receptors on both visceral and vascular smooth muscle to produce contractions (see Burnstock, 1990, 2009b) and ATP released together with acetylcholine (ACh) from parasympathetic nerves acts on P2X1 receptors in the urinary bladder (Burnstock et al., 1978; Burnstock, 2013). ACh acting via nicotinic receptors was established early as the neurotransmitter released from motor nerves supplying adult skeletal muscle, but later it was shown that during postnatal development of the neuromuscular junction, ATP is released as a cotransmitter together with ACh to act on P2X receptors (see Henning, 1997). An important advance was made when purinergic synaptic transmission between nerves was described in both the coeliac ganglion (Evans et al., 1992; Silinsky et al., 1992) and medial habenula in the brain (Edwards et al., 1992).

P2X receptors have also been shown to act presynaptically, for example P2X3 receptors on primary afferent sensory nerve endings in the dorsal spinal cord to enhance the release of glutamate (Gu and MacDermott, 1997) and on P2X receptors on sympathetic nerve varicosities in the vas deferens to enhance the release of NA (Queiroz et al., 2003).

P2X3 homomultimer and P2X2/3 heteromultimer receptors were identified on sensory neurons and nerve endings (Chen et al., 1995; Lewis et al., 1995) mediating both physiological reflex responses as well as nociception (see Burnstock and Verkhratsky, 2012b).

**Figure 1:**
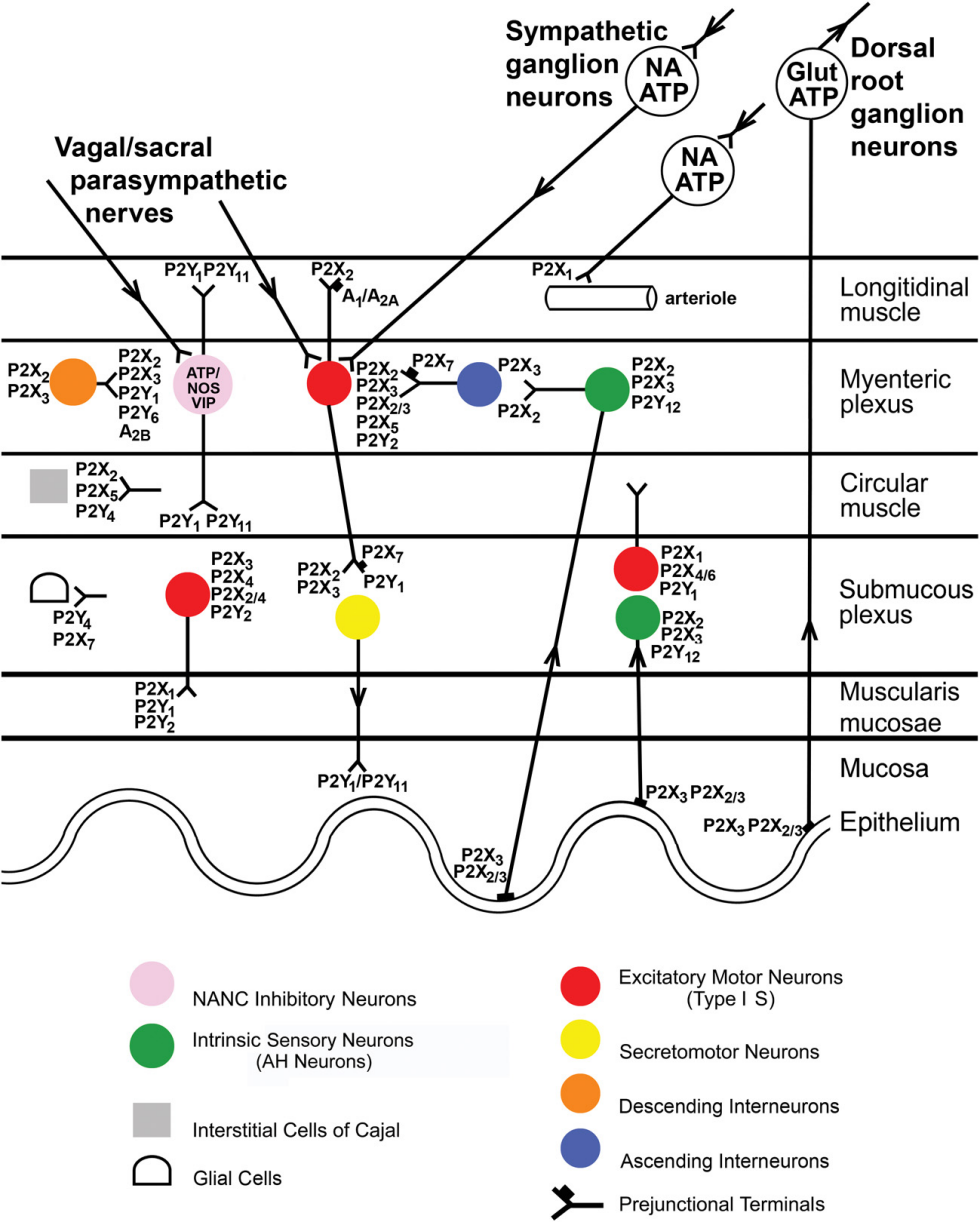
**Distribution of P2X receptor subtypes in the gut.** Extrinsic vagal and sacral parasympathetic nerves connect with NANC inhibitory neurons in the myenteric plexus expressing P2X2 and P2X3 receptors, as well as with cholinergic motor neurons; these neurons are also activated by descending interneurons. Extrinsic sympathetic nerves modulate motility via excitatory motor neurons and constrict blood vessels in the gut via P2X1 receptors. Extrinsic sensory nerves arise from cell bodies in dorsal root ganglia and with subepithelial terminals expressing P2X3 and P2X2/3 receptors and mediate nociception. Intrinsic sensory neurons in both myenteric and submucosal plexuses express P2X2 and P2X3 receptors; they connect with motor pathways involved in peristalsis. Excitatory motor neurons express P2X2, P2X3, P2X2/3, and P2X5 receptors and connect with both interneurons and secretomotor neurons. Interneurons express P2X2 and P2X3 receptors. Enteric glial cells express P2X7 receptors, while interstitial cells of Cajal express P2X2 and P2X5 receptors. P2X7 receptors appear to act as prejunctional modulators of both motor and interneurons. [Modified from Burnstock (2008c), with permission from the BMJ Publishing Group Ltd].

There is a wide distribution of P2X2, P2X3, P2X2/3, P2X4, and P2X7 receptors in the myenteric and submucous plexuses and on intrinsic and extrinsic sensory nerves of the enteric nervous system (see Burnstock, 2008b and Figure 1). These receptors are involved in reflex activities, including modulation of peristaltic reflexes (Bian et al., 2003; Wynn et al., 2003).

Expression of most P2X receptor subtypes have been localized in different regions of the central nervous system (CNS). Sensory nerves in the brain stem expressing P2X3 receptors and P2X2, P2X4, and P2X6 receptors, mostly in the form of heteromultimers, appear to be involved in both neurotransmission and neuromodulation (see Burnstock, 2007b; Burnstock et al., 2011b; Burnstock and Verkhratsky, 2012b; Lalo et al., 2012). The role of P2X7 receptors in the CNS is controversial. Behavioral studies have implicated roles for P2X receptors in memory and learning, sleep, locomotion and feeding (see Burnstock et al., 2011a).

In the heart, P2X1/3/4/5/6 receptor mRNA and protein are expressed in ventricles and P2X1-6 in atria (Hansen et al., 1999) leading to increase in contractility of cardiac myocytes (Shen et al., 2007). P2X receptor subtypes are widely expressed in different sites in the kidney (see Unwin et al., 2003). Preglomerular arterioles express P2X1 receptors, while glomerular mesangial cells express P2X4, P2X5, and P2X7 receptors, and podocytes express P2X1 and P2X7 receptors. Different regions of the kidney tubule are immunoreactive for P2X receptors: P2X1, P2X4, P2X5, and P2X6 on proximal tubules, P2X4 and P2X6 on distal tubules and P2X1, P2X4/6, and P2X5 on collecting ducts (Bailey and Shirley, 2009). In the collecting ducts P2X4 and P2X4/6 receptors are involved in control of sodium transport (Wildman et al., 2009).

Uptake of organic cations is mediated by P2X1 and P2X7 receptors in canine erythrocytes (Stevenson et al., 2009). The role of the P2X1 receptor expressed by platelets is unclear, although in P2X1 knockout mice there is a decreased level of thrombus formation and increased bleeding times (Nurden, 2007). P2X1 receptors have also been claimed to play a role in sensing bacteria (Kälvegren et al., 2010).

The P2X7 receptor is involved in immunomodulation responding to extracellular ATP at sites of inflammation and tissue damage (see Di Virgilio, 2013). P2X1 receptors promote neutrophil chemotaxis and play a significant role in host defense (Lecut et al., 2009). P2X7 receptors mediate cytokine release and chemokine expression via P2X1 and P2X3 receptors in mouse mast cells (Bulanova et al., 2009). P2X7 receptors in human dendritic cells mediate the release of tissue factor-bearing microparticles (Baroni et al., 2007).

Keratinocyte turnover in skin epidermis involves P2X receptors while P2Y_1_ and P2Y_2_ receptors in basal and parabasal layers mediate cell proliferation, P2X5 receptors in the granular layer mediate cell differentiation and P2X7 receptors at the stratum granulosum/stratum corneum border mediate apoptotic cell death (Greig et al., 2003c; Burnstock et al., 2012b). In the endocrine system, the posterior pituitary expresses protein for P2X2 and P2X6 receptors and P2X2, P2X3, P2X4, and P2X7 receptor channels are present on anterior pituitary cells and mediate hormone secretion (Stojilkovic et al., 2010). P2X7 receptors are expressed on osteoblasts, enhancing differentiation and bone formation and also on osteoclasts mediating apoptosis (see Orriss et al., 2010, 2012).

P2X receptors in the special senses mediate a variety of different functions (see Housley et al., 2009; Burnstock and Verkhratsky, 2012b). Nasal epithelium expresses P2X2, P2X5, and P2X7 receptors (Gayle and Burnstock, 2005), P2X1, P2X2, P2X3, and P2X2/3 are prominent receptors in the tongue (Bo et al., 1999) mediating both taste sensation and pain (Rong et al., 2000), and P2X receptors have multiple roles in the eye (see Pintor, 2006) and inner ear (see Housley and Gale, 2010).

## Pathophysiology of P2X receptors

The involvement of P2X receptors is being investigated increasingly in relation to a wide variety of diseases (see Burnstock, 2006a,b, 2007b, 2008a).

### Diseases of special senses

P2X receptors are expressed by various structures in the eye and novel therapeutic strategies are being developed for glaucoma, dry eye, and retinal detachment (Pintor et al., 2003). P2X7 receptors are increased in retinal microvessels early in experimental diabetes. This suggests that purinergic vasotoxicity may play a role in microvascular cell death, characteristic of diabetic retinopathy (Sugiyama et al., 2004).

P2X receptors have been described in the vestibular system (Xiang et al., 1999), in particular on the endolymphatic surface of the cochlear endothelium, an area associated with sound transduction. It has been suggested that ATP may regulate fluid homeostasis, cochlear blood flow, hearing sensitivity and development, and therefore may be useful for the treatment of Ménière's disease, tinnitus, and sensorineural deafness (Housley, 2000). There is upregulation of P2X2 receptors in the cochlear occurs during sustained loud noise. P2X2 receptor expression is also increased in spiral ganglion neurons (Wang et al., 2003).

Purinergic receptors have been described in the nasal mucosa, including the expression of P2X3 receptors on olfactory neurons (Gayle and Burnstock, 2005). The induction of heat-shock proteins by noxious odor damage is prevented by the administration *in vivo* of P2 receptor antagonists (Hegg and Lucero, 2006).

### Diseases of the kidney and urinary tract

Purinoceptors are expressed in different regions of the nephron, the glomerulus, and renal vascular system in the kidney and different subtypes are involved in the regulation of renin secretion, glomerular filtration and the transport of water, ions, nutrients and toxins (Unwin et al., 2003). Autocrine purinergic signaling enhances cyst expansion and accelerates progression of polycystic kidney disease (Schwiebert et al., 2002). P2X7 receptor expression is increased in cystic tissue from a rat model of autosomal dominant polycystic kidney disease (Turner et al., 2004). Increased glomerular expression of P2X7 receptors has been reported in rat models of glomerular injury due to diabetes and hypertension (Vonend et al., 2004). Human and experimental glomerulonephritis also showed increase in P2X7 receptor expression in the glomerulus (Turner et al., 2007).

P2X3 receptors are expressed by the suburothelial sensory nerves, and both the human and guinea-pig ureter urothelial cells release ATP in a pressure-dependent fashion when the ureter is distended (Knight et al., 2002; Calvert et al., 2008). P2X3 antagonists may be useful to alleviate renal colic (Rong and Burnstock, 2004).

Atropine will block at least 95% of parasympathetic nerve-mediated contraction in the healthy human bladder, showing neurotransmission that is predominantly cholinergic, although P2X1 receptors are present on the smooth muscle (Burnstock, 2001a). However, the purinergic component of parasympathetic cotransmission is increased in pathological conditions (see Burnstock, 2013). It is increased to 40% in interstitial cystitis, outflow obstruction, idiopathic detrusor instability and most types of neurogenic bladder. Release of ATP from distended bladder urothelial cells in patients with interstitial cystitis is significantly greater than from healthy cells (Tempest et al., 2004) and P2X1 receptor subtype expression is increased in obstructed bladder (Boselli et al., 2001).

Purinergic signaling also plays a role in afferent sensation from the bladder, involved in both the micturition reflex and pain. Release of ATP from urothelial cells occurs during distension (Vlaskovska et al., 2001) and it acts on P2X3 receptors on suburothelial sensory nerve endings (Cockayne et al., 2000). P2X3 receptors are therefore a potential target for pharmacological manipulation in the treatment of both pain and detrusor instability. In idiopathic detrusor instability, there is abnormal purinergic transmission in the bladder (O'Reilly et al., 2002). Voiding dysfunction involves P2X3 receptors in conscious chronic spinal cord injured rats, suggesting that P2X3 antagonists might also be useful for the treatment of neurogenic bladder (Lu et al., 2002). Drugs that alter ATP release or breakdown might also be considered as therapeutic targets (Chess-Williams, 2004). A recent review about purinergic signaling in the lower urinary tract is available (Burnstock, 2013).

### Cardiovascular diseases

There is up-regulation of P2X1 receptor mRNA in the hearts of rats with congestive heart failure and an increase in expression of P2X1 receptors in the atria of patients suffering from dilated cardiomyopathy. P2X4 receptor mRNA was reported to be upregulated in ligation-induced heart failure and was claimed to have a beneficial life-prolonging role (Musa et al., 2009).

ATP, released as the purinergic component of sympathetic cotransmission, is increased in spontaneously hypertensive rats mediating vasoconstriction via P2X1 receptors (see Ralevic and Burnstock, 1998). There is upregulation of placental P2X4 receptors in mild preeclampsia (Roberts et al., 2007).

### Disorders of the gut

P2X receptors play major roles in diseases of the gut (see Burnstock, 2008a,b). P2X7 receptors, that mediate cytokine production, may play a role in the response of enteric glia to inflammation (Vanderwinden et al., 2003). Enhancement of P2X3 receptor-mediated purinergic signaling in an animal model of colitis has been described (Wynn et al., 2004). P2X3 receptor expression is also increased in the enteric plexuses in human irritable bowel syndrome (IBS), suggesting a role in dysmotility and pain initiation (Yiangou et al., 2001; Galligan, 2004; Shinoda et al., 2009). Visceral hyperalgesia induced in a rat model of IBS was associated with potentiation of ATP-evoked responses and an enhanced expression of P2X3 receptors in sensory neurons in the colon (Xu et al., 2008). In aganglionic bowel from Hirschsprung's disease patients, P2X3 immunohistochemistry was demonstrated, suggesting that the sensory nerves may be involved (Facer et al., 2001).

Both intrinsic sensory neurons in the submucous plexus of the gut and extrinsic sensory nerves with cell bodies in the dorsal root ganglia (DRG), show positive immunoreactivity for P2X3 receptors (Xiang and Burnstock, 2004). It has been suggested that during moderate distension, low threshold intrinsic enteric sensory fibers are activated, via P2X3 receptors, by ATP released from mucosal epithelial cells resulting in reflexes concerned with propulsion of material down the gut (Burnstock, 2001b). Peristalsis is impaired in the small intestine of mice lacking the P2X3 receptor subunit, which supports this view (Bian et al., 2003). During substantial (colic) distension associated with nociception, higher threshold extrinsic sensory fibers may be activated by ATP released from the mucosal epithelial cells to pass messages through the DRG to pain centers in the CNS (Wynn et al., 2003, 2004). Sensitization of P2X3 receptors on vagal and spinal afferents in the stomach have been claimed to contribute to dyspeptic symptoms and to the development of visceral hyperalgesia (Dang et al., 2005). A recent review describing P2X receptors in the gut is available (Burnstock, 2012).

### Diseases of the reproductive system

ATP induces a significant increase in sperm fertilizing potential and this has led to the use of ATP for treatment of spermatozoa during *in vitro* fertilization (Rossato et al., 1999). P2X1 receptor knockout mice appear normal, but fail to breed and this is associated with loss of the purinergic component of sympathetic cotransmission in the vas deferens (Dunn, 2000; Mulryan et al., 2000). P2X receptor subtypes are expressed at different stages during spermatogenesis in the adult rat testis, which may be novel targets for both fertility and contraception (Glass et al., 2001).

Low concentrations of ATP stimulate changes in transepithelial conductance in the human uterine cervix, the first phase mediated by P2Y_2_ receptors and the second phase by P2X4 receptors (Gorodeski, 2002).

### Diabetes

There is an enhancement of P2X7 receptor-induced pore formation and apoptosis in early diabetes in the retinal microvasculature (Sugiyama et al., 2004). P2X7 receptors are located on glucagon-containing α cells in pancreatic islets (Coutinho-Silva et al., 2001). In streptozotocin-diabetic rats P2X7 receptor-labeled α cells migrate centrally to take the place of the insulin-containing β cells, although the functional significance of this is unknown (Coutinho-Silva et al., 2003). Central neuropathic complications occur in diabetic neuropathy, including decreased cognitive performance and it has been shown that synaptic ATP signaling is depressed in streptozotocin-induced diabetic rats (Duarte et al., 2007). The density of P2X3/5/7 receptors was decreased in the hippocampal nerve terminals of diabetic rats. A recent review of the literature concerned with purinergic signaling in diabetes is available (Burnstock and Novak, 2013).

### Diseases of the airways

Lung epithelial cells express P2X4 receptors that are involved in regulation of ciliary beat, manipulation of which may be of therapeutic benefit for cystic fibrosis (Zsembery et al., 2003). Vagal afferent purinergic signaling may be involved in the hyperactivity associated with asthma and chronic obstructive pulmonary disease (Adriaensen and Timmermans, 2004). Erythromycin, used for the treatment of upper and lower respiratory tract infections, blocks P2X receptor-mediated Ca^2+^ influx and may be involved in its anti-secretory effects in the treatment of chronic respiratory tract infections (Zhao et al., 2000).

A network of respiratory neurons in the ventrolateral medulla (VLM) is responsible for the generation of the respiratory rhythm and also functions as a chemoreceptive area mediating the ventilating response to hypercapnia. ATP acting via P2X2 receptors expressed on VLM neurons is involved in these functions (Gourine et al., 2003). P2 receptor synaptic signaling in respiratory motor control has been implicated by the multiple physiological effects of ATP in hypoglossal activity mediated by P2X2, P2X4, and P2X6 receptors in the nucleus ambiguous and the hypoglossal nucleus (Collo et al., 1996). ATP injected into the caudal nucleus of the solitary tract of awake rats produced respiratory responses (Antunes et al., 2005).

P2X7 receptors are expressed in alveolar macrophages, which play a pivotal role in the development of chronic lung inflammatory reactions, such as idiopathic pulmonary fibrosis, silicosis, asbestosis, hypersensitivity pneumonitis, sarcoidosis and mycobacterium tuberculosis (Lemaire and Leduc, 2004). Stimulation of P2X7 receptors results in activation of the proinflammatory interleukin (IL)-1 to IL-5 cytokine cascade and the formation of multinucleated giant cells, a hallmark of granulomatous reactions. A recent review describing purinergic signaling in the airways in health and disease has been published recently (Burnstock et al., 2012a).

### Diseases of skin

An increase of P2X3 and P2X2/3 nociceptive receptors on sensory nerve endings in inflamed skin has been reported and antagonists are being explored as analgesics (Hamilton et al., 2001). A pathogenic role for keratinocyte-derived ATP in irritant dermatitis has been suggested (Mizumoto et al., 2003). There are changes in expression of purinergic receptors in the regenerating epidermis in wound healing (Greig et al., 2003a). Acceleration of skin barrier repair and prevention of epidermal hyperplasia induced by skin barrier disruption by P2X receptor antagonists has been reported (Denda et al., 2002). A review about purinergic signaling in skin in health and disease is available (Burnstock et al., 2012b).

### Immune system and inflammation

P2X7 receptors expressed by inflammatory and immune cells play a pivotal role in inflammation and immunomodulation (Di Virgilio, 2007, 2013). The treatment of neurogenic inflammation, rheumatoid arthritis, and periodontitis by purinergic compounds is being explored. P2X7 receptor-mediated apoptosis in macrophages results in killing of the mycobacteria contained within them, unlike the macrophage apoptosis produced by other agents (Lammas et al., 1997). There is accumulation of macrophages expressing P2X4 receptors in rat CNS lesions during experimental autoimmune encephalomyelitis (Guo and Schluesener, 2005). It has been suggested that ATP may be mechanistically involved in human allergic/asthmatic reactions (Schulman et al., 1999). P2X7 receptors are expressed by alveolar macrophages, which, when activated, trigger pro-inflammatory activation of IL1-6 cytokines and granulomatous reactions (Lemaire and Leduc, 2004). A lower concentration of ATP activation of P2X7 receptors can result in cell proliferation (Di Virgilio et al., 2009). The functional expression of P2X7 receptors on B lymphocytes may be related to the severity of B-cell chronic lymphocytic leukaemia (Adinolfi et al., 2002).

ATP induces cell death in CD4^+^/CD8^+^ double-positive thymocytes during the acute phase of *Trypanosoma cruzi* infection in Chaga's disease and may play a role in the thymus atrophy that occurs in Chaga's disease (Mantuano-Barradas et al., 2003). *Schistosoma mansoni*, a parasitic blood fluke, also produces thymic atrophy, and the P2X receptor cloned from *S. mansoni* provided an example of a non-vertebrate ATP-gated ion channel and suggests a drug target for the treatment of schistosomiasis (Agboh et al., 2004).

### Cancer

The use of adenine nucleotides as anticancer agents was first described by Rapaport (1983). ATP, injected intraperitoneally into tumor-bearing mice, resulted in anticancer activity against several fast-growing aggressive carcinomas (Agteresch et al., 2003). Evidence has been presented that extracellular ATP inhibits the growth of a variety of human tumors, including prostate, bladder, breast, colon, liver, ovarian, colorectal, oesophageal and melanoma cancer cells, partly via P2X7 receptors mediating apoptotic cancer cell death (Abraham et al., 2003; White and Burnstock, 2006). Studies have been carried out to determine the P2 receptor subtypes that contribute to ATP suppression of malignant melanomas (White et al., 2005a,b), basal and squamous cell tumors (Greig et al., 2003b) and prostate and bladder cancers (Calvert et al., 2004; Shabbir et al., 2008a,b). P2X5 receptors mediate cell differentiation, which in effect is antiproliferative and apoptotic cell death is mediated by P2X7 receptors. A review has been published recently entitled “Purinergic signaling and cancer” (Burnstock and Di Virgilio, 2013).

### Musculoskeletal diseases

Purinergic signaling is involved in bone development and remodeling (Hoebertz et al., 2003; Burnstock and Arnett, 2006; Orriss et al., 2010). Osteoclasts, osteocytes, osteoblasts and chondrocytes all express P2X receptors. Regulatory roles in bone formation and resorption by P2X7 receptors were revealed by studies of P2X7 receptor knockout mice. The purinoceptors on bone and cartilage represent potential targets for the development of novel therapeutics to inhibit bone resorption in musculoskeletal diseases, including rheumatoid arthritis, osteoporosis, tumor-induced osteolysis, and periodontitis (Komarova et al., 2001). The P2X7 receptor antagonist, oxidized ATP, reduced inflammatory pain in arthritic rats (Dell'Antonio et al., 2002).

Lymphoblastoid cells from Duchenne muscular dystrophy patients are sensitive to stimulation by extracellular ATP (Ferrari et al., 1994). Evidence has been presented for a role for P2X receptor-mediated signaling in muscle regeneration using the *mdx* mouse model of muscular dystrophy, which raised the possibility of new therapeutic strategies for the treatment of muscle disease (Ryten et al., 2004). A recent review about purinergic signaling in the musculoskeletal system is available (Burnstock et al., 2013).

### Disorders of the central nervous system

Recent reviews have focused on purinergic signaling in disorders of the CNS (Burnstock, 2008a; Burnstock et al., 2011a; Franke et al., 2012; Volonté and Burnstock, 2012).

Microglia and macrophages expressing P2X4 receptors accumulate following experimental traumatic brain injury and spinal cord injury. Activated microglia also show increase in P2X7 receptor expression, which initiate microglial proliferation and death. Lesions in the cerebellum result in upregulation of P2X1 and P2X2 receptors in precerebellar nuclei, and there is increased expression of several subtypes of P2X receptors after stab wound injury in the nucleus accumbens (Franke et al., 2006). P2X7 receptors are upregulated following ischaemia on neurons and glial cells in rat cerebral cortex, and become supersensitive in cerebrocortical cell cultures (Cavaliere et al., 2003). Ischaemic cell death was prevented by P2 receptor antagonists.

Involvement of P2X receptors in neurodegenerative diseases such as Parkinson's, Alzheimer's, Huntington's, amyotrophic lateral sclerosis (ALS) and multiple sclerosis (MS) has been described (see Burnstock, 2008a). In the pathogenesis of Parkinson's disease, release of ATP from disrupted cells may cause cell death in neighboring cells expressing P2X7 receptors, leading to a necrotic volume increase. Upregulation of P2X7 receptors in human Alzheimer's diseased brains and in animal models has been reported (Parvathenani et al., 2003; McLarnon et al., 2006) and stimulation of P2X7 receptors on human microglia and macrophages increased the degenerative lesions observed in Alzheimer's disease. In two different transgenic models of Huntington's disease, changes in P2X receptor-mediated neurotransmission in cortico-striatal projections were observed (Diez-Zaera et al., 2007). Both P2X4 and P2X7 receptors have been implicated in the transgenic superoxide dismutase 1 (SOD1) mouse model of ALS (Andries et al., 2007; Apolloni et al., 2013). In MS lesions in brain tissue, P2X7 receptors were detected on reactive astrocytes (Narcisse et al., 2005). Lesional accumulation of P2X receptors on macrophages in the CNS of the rat model of MS, experimental autoimmune encephalomyelitis, has been reported (Guo and Schluesener, 2005). P2X7 expression is elevated in astrocytes in MS patients (Narcisse et al., 2005).

P2X7 receptors on microglia, the immune cells in the CNS, are activated by purines to release inflammatory cytokines such as IL-1β, IL-6, and tumor necrosis factor-α (Di Virgilio, 2007). P2X7 receptors have been implicated in the formation of multinucleated giant macrophage-derived cells, a feature of chronic inflammatory reactions (Lemaire et al., 2006). Prion infection has been claimed to be associated with hypersensitivity of P2X7 receptors in microglia (Takenouchi et al., 2007). Microglial cell activation by pro-inflammatory bacterial lipopolysaccharide leads to a transient increase in ivermectin-sensitive P2X4 receptor currents (Raouf et al., 2007). Activation of astrocytes via P2X7 receptors increases chemokine monocyte chemoattractant protein-1 expression and it was suggested that this may be important for communication with haematopoietic inflammatory cells (Panenka et al., 2001).

Generalized motor seizures can be evoked by microinjection of ATP analogs into the prepiriform cortex (Knutsen and Murray, 1997). The prepiriform cortex expresses P2X2, P2X4, and P2X6 receptors and it was suggested that P2X receptor antagonists may have potential as neuroleptic agents. In chronic epileptic rats, the hippocampus showed abnormal responses to ATP, associated with increased expression of P2X7 receptors, which were upregulated in rats with chronic pilocarpine-induced epilepsy and may be involved in the pathophysiology of temporal lobe epilepsy. Enhanced immunoreactivity of the P2X7 receptor was observed in microglia from rat brain following kainate-provoked seizures (Rappold et al., 2006). A decrease of presynaptic P2X receptors in the hippocampus of rats that have suffered a convulsive period has been shown (Oses, 2006). Glutamate release from astrocytes induced by ATP has been described in epileptogenesis (Tian et al., 2005).

The P2X7 receptor gene has been implicated in both major depressive illness (Lucae et al., 2006) and bipolar affective disorders (Barden et al., 2006). In schizophrenia, the involvement of ATP receptors has been implicated in relation to reports that the antipsychotic drugs haloperidol, chlorpromazine and fluspirilene, inhibit ATP-evoked responses mediated by P2X receptors (Inoue et al., 1996). It has been suggested that ATP may have a facilitating role for dopaminergic transmission and that some antipsychotic drugs express their therapeutic effects by suppression of dopaminergic hyperactivity through inhibition of P2X receptor-mediated effects. Ethanol is probably the oldest and most widely used psychoactive drug. The cellular mechanisms underlying its actions are not well-understood, but some insights in relation to purinergic P2 receptor signaling have emerged in recent years (Davies et al., 2005). P2X receptor-mediated responses of DRG neurons are inhibited by ethanol by an allosteric mechanism. For P2X4 receptors, ethanol inhibition is altered by mutation of histidine 241. Ethanol differentially affects ATP-gated P2X3 and P2X4 receptor subtypes expressed by *Xenopus* oocytes.

### Pain

There are reviews that have addressed this topic (see, for example, Burnstock, 2009c,d; Jarvis, 2010; Tsuda et al., 2010; Trang et al., 2012). Visceral pain is a common form of pain associated with pathological conditions such as renal colic, dyspepsia, inflammatory bowel disease, angina, dysmenorrhoea, and interstitial cystitis. P2X3 (homomultimer) and P2X2/3 (heteromultimer) receptors have been cloned and shown to be mainly located on small nociceptive sensory neurons in the DRG (Lewis et al., 1995).

**Figure 2:**
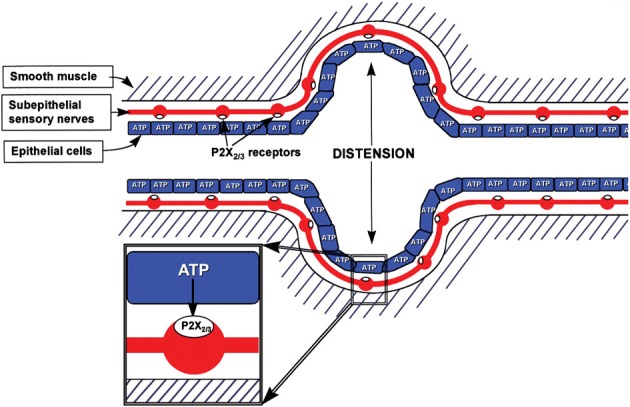
**Schematic representation of hypothesis for purinergic mechanosensory transduction in tubes (e.g., ureter, vagina, salivary and bile ducts, gut) and sacs (e.g., urinary and gall bladders, lung).** It is proposed that distension leads to release of ATP from epithelium lining the tube or sac, which then acts on P2X_3_ and/or P2X_2/3_ receptors on subepithelial sensory nerves to convey sensory/nociceptive information to the CNS. [Reproduced from Burnstock (1999), with permission from Wiley].

It was proposed in 1999 that purinergic mechanosensory transduction occurred in visceral tubes and sacs, including ureter, bladder and gut, where ATP released from lining epithelial cells during distension acted on P2X3 and P2X2/3 receptors on subepithelial nociceptive sensory nerves to initiate impulses in sensory pathways to pain centers in the CNS (Burnstock, 1999) (Figure 2). P2X3 receptor knockout mice exhibited reduced inflammatory pain and marked urinary bladder hyporeflexia with reduced voiding frequency, suggesting that P2X3 receptors were involved in mechanosensory transduction underlying both inflammatory pain and physiological voiding reflexes (Cockayne et al., 2000). ATP was shown to be released from bladder urothelial cells during distension, and activity initiated in pelvic sensory nerves was mimicked by ATP and α,β-methylene ATP (α,β-meATP) and attenuated by P2X3 antagonists as well as in P2X3 knockout mice (Vlaskovska et al., 2001). Passage of a kidney stone through the ureter causes severe pain. P2X3 receptor immunostaining of sensory nerves in the suburothelial region was reported (Lee et al., 2000). Using a guinea-pig preparation, perfused *in vitro*, multifiber recordings of ureter afferent nerve activity were made (Rong and Burnstock, 2004). Distension of the guinea-pig ureter resulted in increased spike discharge in sensory nerves, which was mimicked by ATP and reduced by P2X3 receptor antagonists. Pressure-dependent release of ATP from urothelial cells to about 10 times the basal release levels resulted from distension of both the perfused guinea-pig and human ureters (Knight et al., 2002; Calvert et al., 2008).

Purinergic mechanosensory transduction in the gut initiated both physiological reflex modulation of peristalsis via intrinsic sensory fibers and nociception via extrinsic sensory fibers (Burnstock, 2001b). Distension of a pelvic sensory nerve-colorectal preparation led to pressure-dependent increase in release of ATP from mucosal epithelial cells and evoked pelvic nerve excitation. This excitation was mimicked by application of ATP and α,β-meATP and attenuated by selective P2X3 and P2X2/3 antagonists (Wynn et al., 2003).

P2X3 and P2X2/3 receptors located on primary afferent nerve terminals in inner lamina 2 of the spinal cord, also play a significant role in neuropathic and inflammatory pain (see Wirkner et al., 2007; Burnstock, 2009a). Dorsal horn neurons relaying nociceptive information further along the pain pathway express P2X2, P2X4, and P2X6 receptors (Bardoni et al., 1997). Microglial P2X4 and P2X7 receptors are also involved in neuropathic pain (Tsuda et al., 2003; Hughes et al., 2007), although the underlying mechanisms are still under investigation (Inoue, 2007; Trang and Salter, 2012). Neuropathic pain and allodynia are abolished in both P2X4 and P2X7 knockout mice, so there is much interest in finding selective antagonists that are suitable for therapeutic development (see McGaraughty et al., 2007).

ATP involvement in migraine was first suspected in relation to the vascular theory of this disorder with ATP released from endothelial cells in microvessels during reactive hyperaemia, which is associated with pain, following cerebral vascular vasospasm (that is not associated with pain; Burnstock, 1989). P2X3 receptor involvement in neuronal dysfunction in brain areas that mediate nociception in migraine, such as the trigeminal nucleus and thalamus, has also been proposed (Fabbretti et al., 2006), and may represent a novel target for antimigraine drugs (Fumagalli et al., 2006). Anti-nerve growth factor treatment suppressed responses evoked by P2X3 receptor activation in an *in vivo* model of mouse trigeminal pain (D'Arco et al., 2007).

## P2X receptor agonists and antagonists—therapeutic potential

P2X receptors consist of a family of ligand-gated cation channels that are widely expressed in nerves and many non-neuronal cells. Table 2 summarizes the selective agonists and antagonists currently available for the P2X receptor subtypes. With the recent discovery of their crystal structure (Kawate et al., 2009), medicinal chemists now have a detailed understanding of how the individual subunits that form the receptor interact with each other and are in a better position to prepare selective P2X receptor agonists and antagonists. P2X receptors change expression in pathological conditions, suggesting that they may be useful targets for treatment of diseases. The clinical manipulation of purinergic signaling is in its infancy. One of the main reasons why we do not yet have more purinergic therapeutic drugs is the scarcity of receptor-subtype-selective agonists and antagonists that can be used *in vivo*. Afferent Pharmaceuticals have recently developed some small molecules (AF-353 and derivatives) as P2X3 and P2X2/3 antagonists that are orally bioavailable and stable *in vivo* and which are currently in clinical trial (Gever et al., 2006, 2010). There has also been promising development of clinically relevant P2X7 antagonists recently, notably the Abbott compounds A438079 and A-317491 (McGaraughty et al., 2007). However, antagonists for some of the other P2X subtypes are still to be developed. Therapeutic strategies in the future are also likely to include agents that control the expression of P2 receptors, inhibitors of extracellular breakdown of ATP and enhancers and inhibitors of ATP transport.

**Table 2:** **Agonists and antagonists for the different P2X receptor subtypes**.

**Receptor Subtype**	**Agonists**	**Antagonists**
P2X1	BzATP > ATP = 2-MeSATP =α,β-meATP = L-β,γ-meATP (rapid desensitization); PAPET-ATP	NF449 > IP_5_I > TNP-ATP > RO 0437626 > NF279, NF023, RO1, MRS2159
P2X2	ATP ≥ ATPγS ≥ 2-MeSATP >>α,β-meATP (pH + zinc sensitive); β,γ-CF_2_ATP	PSB-1011 > RB2, isoPPADS > PPADS > Suramin, NF770, NF778, Aminoglycoside
P2X3	2-MeSATP ≥ ATP ≥ Ap_4_A ≥ α,β-meATP (rapid desensitization); PAPET-ATP; BzATP	TNP-ATP, isoPPADS > A317491 > NF110 > PPADS, Ip_5_I, phenol red, RO4, RN-1838, Spinorphin, AF353
P2X4	ATP >>α,β-meATP >> CTP, 2-MeSATP Ivermectin potentiation	5-BDBD >> TNP-ATP, PPADS > BBG, Paroxetine, phenolphthalein, CO donor (CORM 2)
P2X5	ATP = 2-MeSATP = ATPγS >>α,β-meATP > AP_4_A	BBG > PPADS, Suramin
P2X6	- (only functions as a heteromultimer)	–
P2X7	BzATP > ATP ≥ 2-MeSATP >>α,β-meATP	KN62, BBG, KN04, MRS2427, O-ATP, RN-6189, AZ10606120, A740003, A-438079, A-804598, GSK-1370319, Compound 31 (GSK), AZD-9056, CE-224,535

## Topics covered in this special issue

Included in this Special Issue are papers by Elsa Fabbretti, Rashid Giniatullin and Anthony Ford about P2X3 receptors; Stanko Stojilkovic, Terrance Egan, Ruth Murell-Lagnado, Annette Nicke, Thomas Grutter and Philippe Seguela about the molecular physiology and targeting of P2X receptors; Sam Fountain about the evolution of P2X receptors; Manfred Frick and Kazu Inoue about P2X4 receptors involved in lung surfactant secretion and microglia-mediated neuropathic pain; David Henshall about P2X receptors as therapeutic targets for epilepsy; Gary Housley and Sue Kinnamon about P2X receptors in hearing and taste; and Antony Triller about P2X7 receptors.

### Conflict of interest statement

The author declares that the research was conducted in the absence of any commercial or financial relationships that could be construed as a potential conflict of interest.
